# Supplementary material for: Higher-order interactions shape microbial interactions as microbial community complexity increases
Source: Sci Rep. 2022 Dec 31;12:22640. doi: 10.1038/s41598-022-25303-1 (PMC9805437; doi:10.1038/s41598-022-25303-1)
Supplement: Supplementary file 5 — Supplementary Figures. [file 41598_2022_25303_MOESM5_ESM.pdf]

SUPPLEMENTARY FIGURES

Higher-order interactions shape microbial interactions as microbial community complexity increases

Manon A. Morin[1*], Anneliese J. Morrison[2,3], Michael J. Harms[2,3], and Rachel J. Dutton[1*]

[1] School of Biological Science, University of California San Diego, La Jolla, 92093, USA

[2] Department of Chemistry and Biochemistry, University of Oregon, Eugene, OR, United States

[3] Institute of Molecular Biology, University of Oregon, Eugene, OR, United States

[*] manon.morin@ymail.com; rjdutton@ucsd.edu

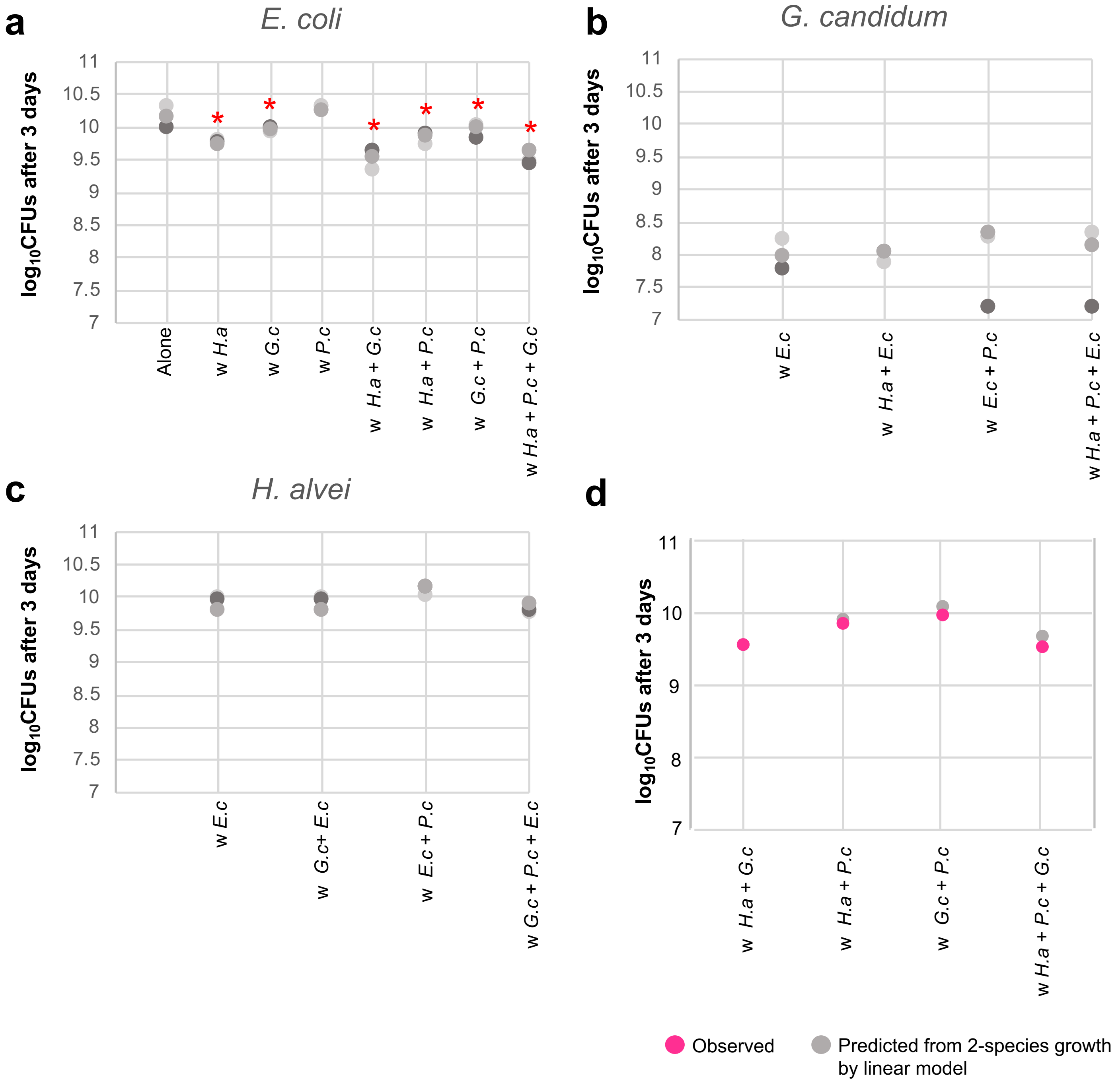

<u>Supplementary Figure 1: Growth of the community species in the studied conditions.</u>

Here, we show the $\log_{10}$ of CFUs for each species in the different conditions for each replicate after 3 days of growth. Dark gray: Replicate 1; Medium gray: Replicate 2; Light grey: Replicate 3

E.c: E. coli; H.a: H. alvei; G.c: G. candidum; P.c: P. camemberti

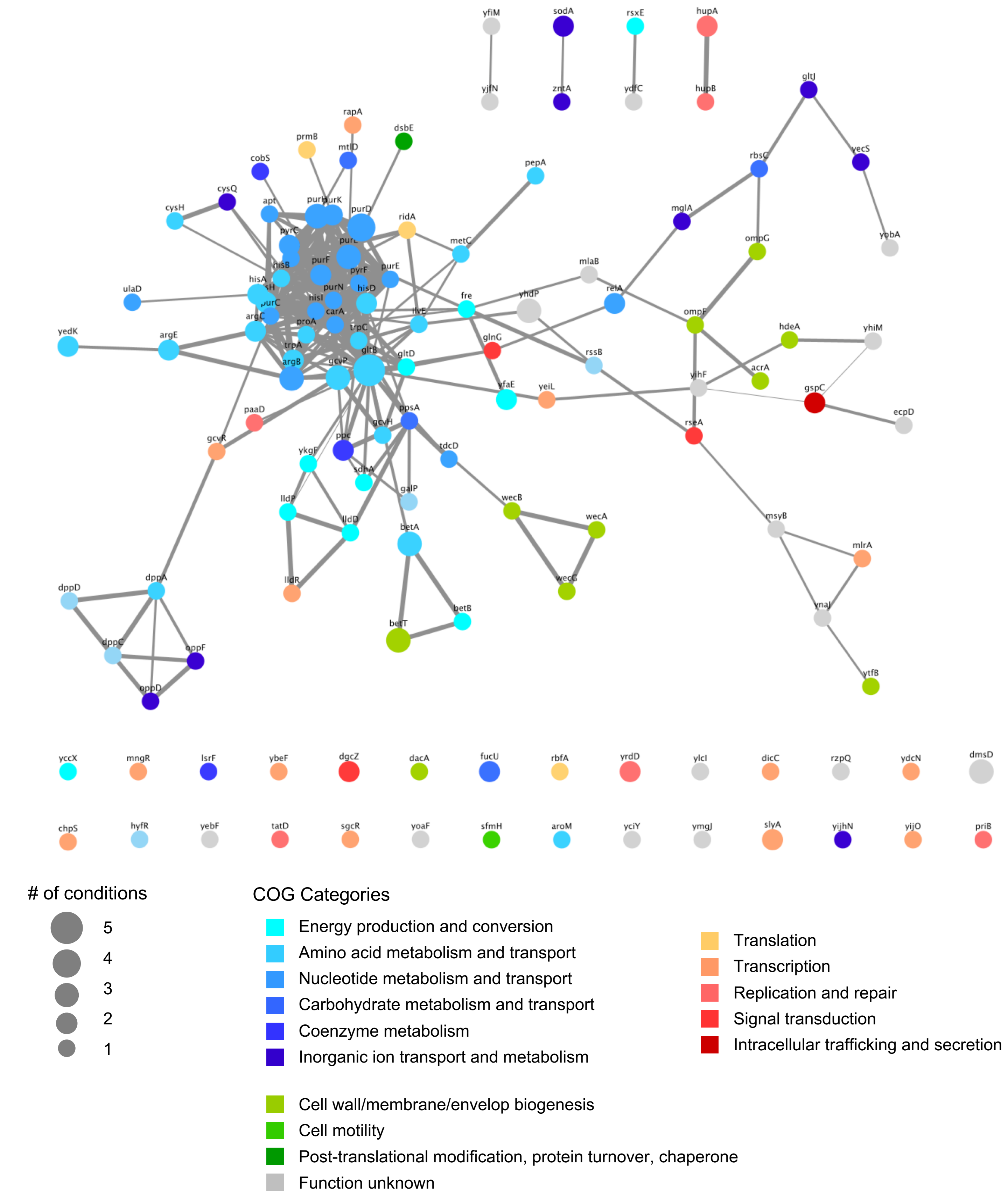

<u>Supplementary figure 2: Functional analysis of genes associated with interaction-associated mutants with negative IFEs.</u> STRING network of the genes associated with negative IFEs (Nodes). Edges connecting the genes represent both functional and physical protein association and the thickness of the edges indicates the strength of data support (minimum required interaction score: 0.4 – medium confidence). Nodes are colored based on their COG annotation and the size of each node is proportional to the number of interactive conditions in which that given gene has been found associated with a significant IFE.

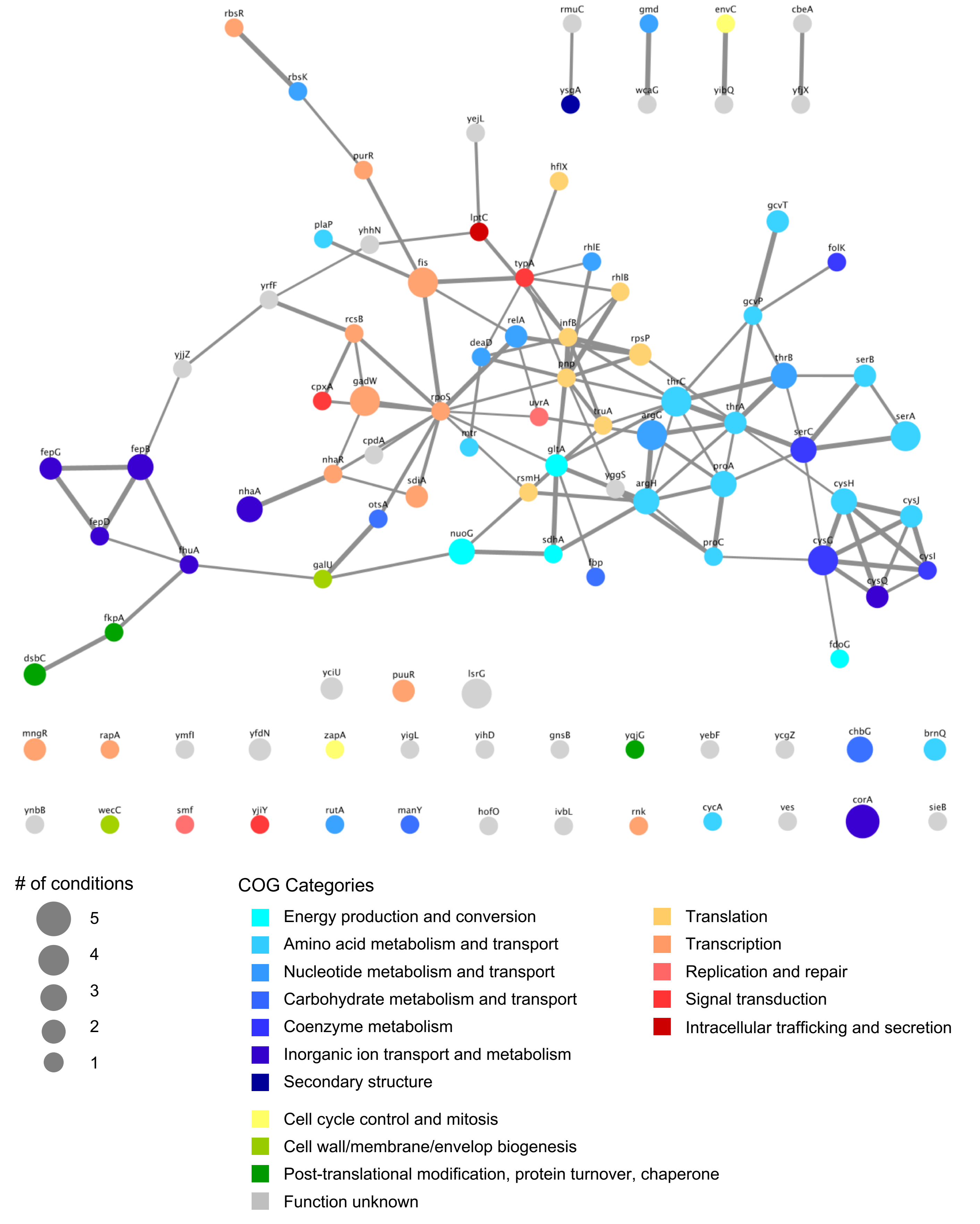

Supplementary figure 3: Functional analysis of genes associated with interaction-associated mutants with positive IFEs. STRING network of the genes associated with positive IFEs (Nodes). Edges connecting the gens represent both functional and physical protein association and the thickness of the edges indicates the strength of data support (minimum required interaction score: 0.4 – medium confidence). Nodes are colored based on their COG annotation and the size of each node is proportional to the number of interactive conditions in which that given gene has been found associated with a significant IFE.

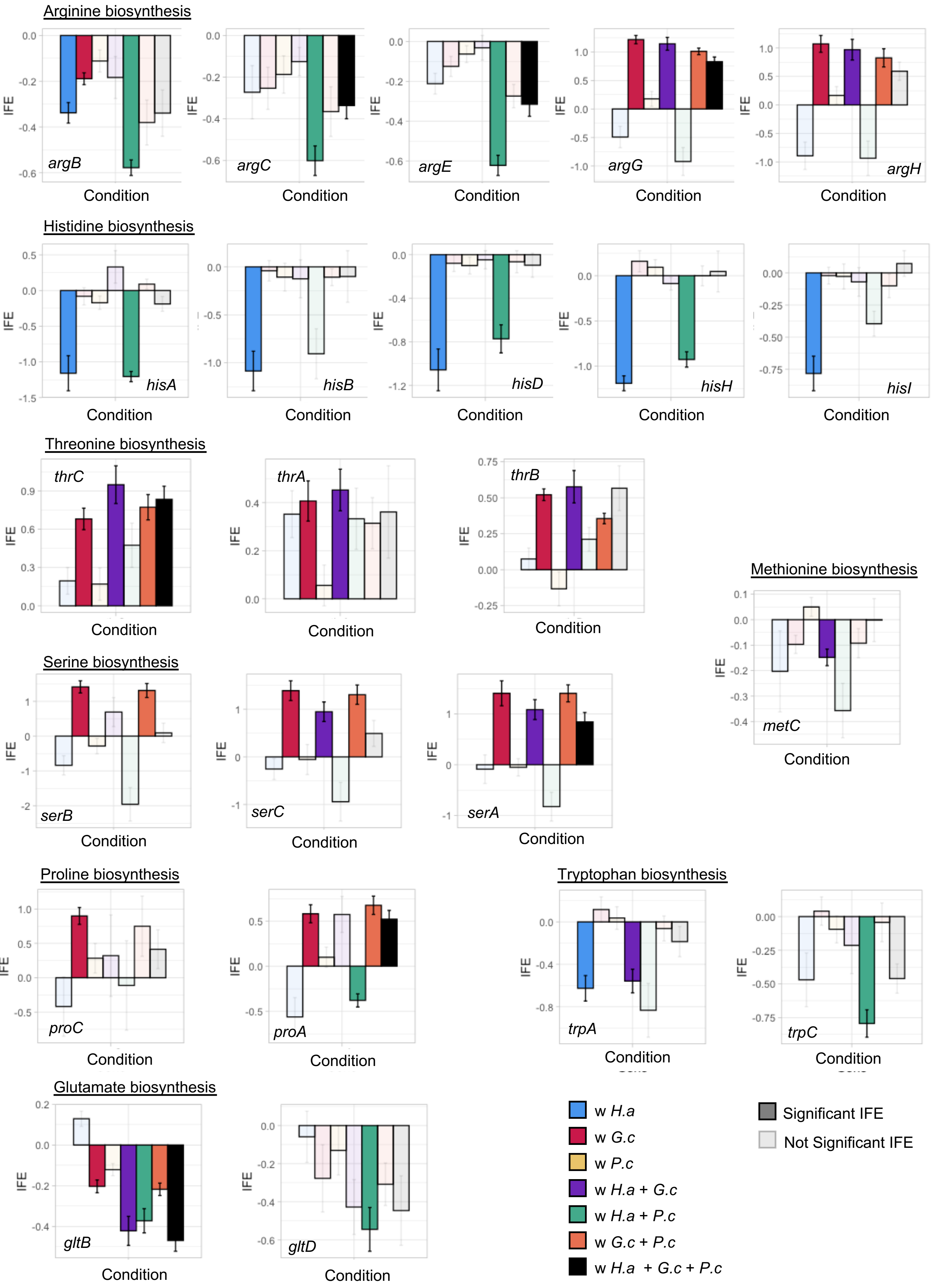

Supplementary figure 4: IFE profiles of Amino acid biosynthesis genes identified in this study

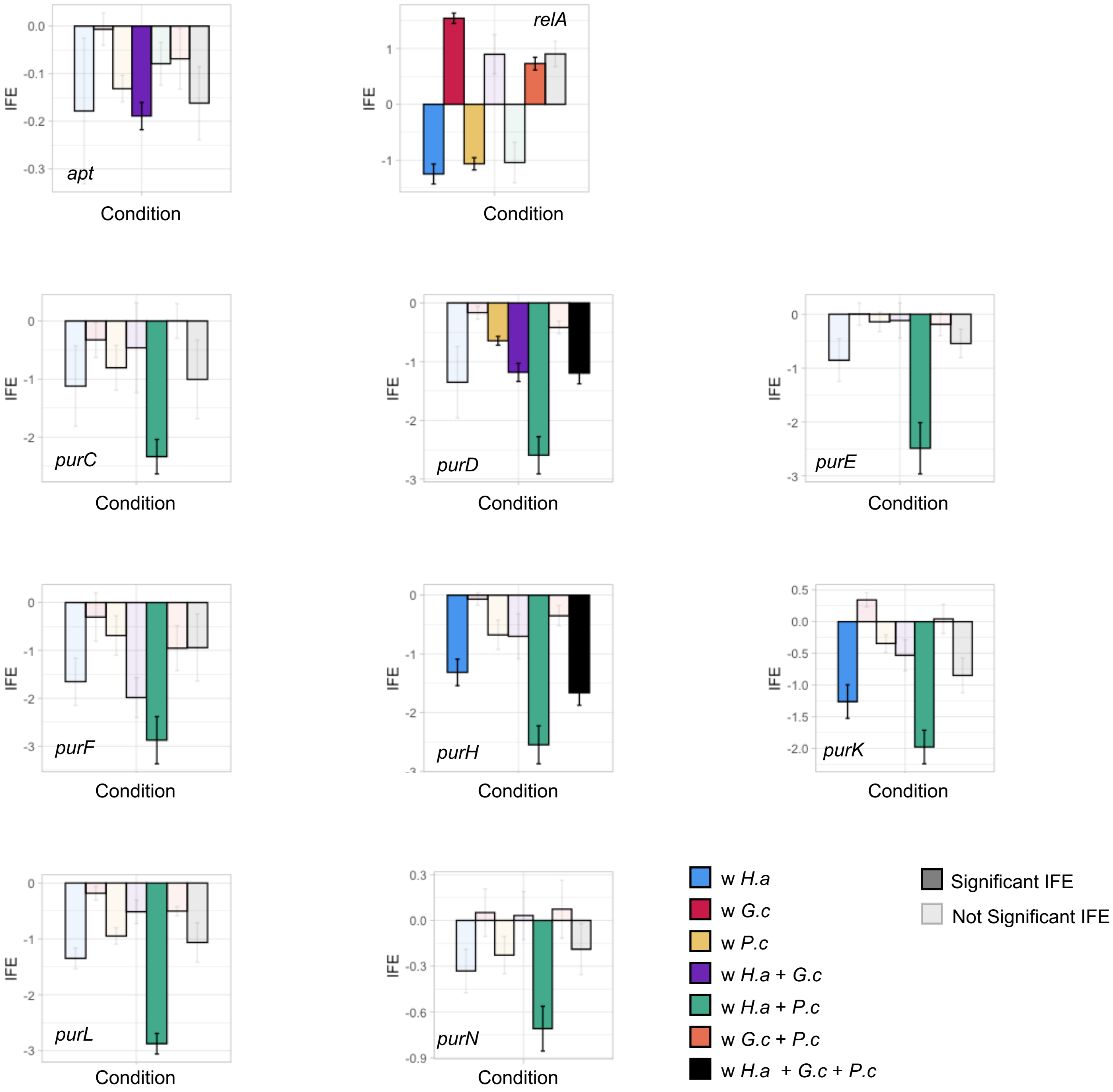

Supplementary figure 5: IFE profiles of Purine biosynthesis associated genes identified in this study

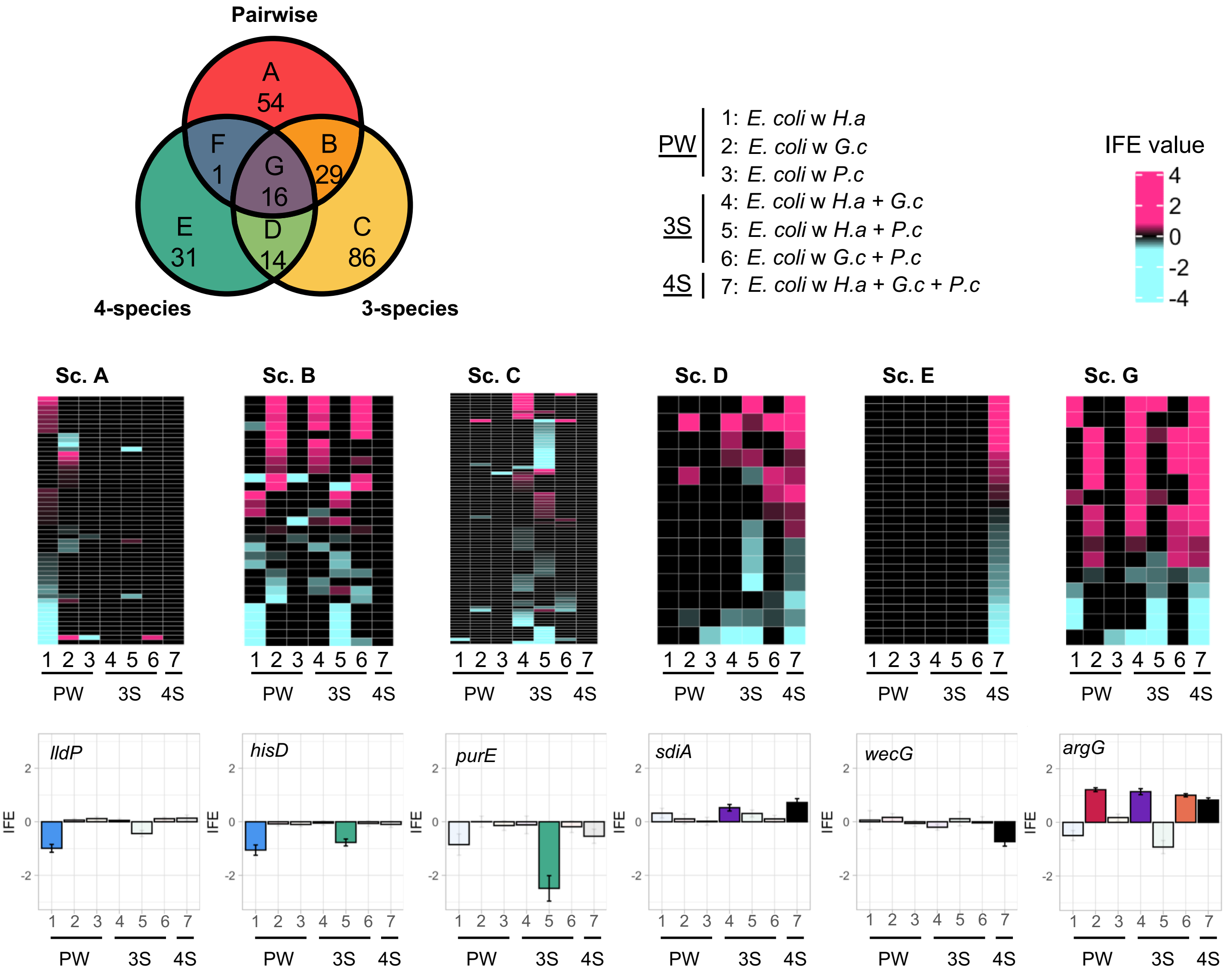

Supplementary Figure 6: Comparison of interaction-associated mutants across the different levels of community complexity.

The Venn Diagram identifies 7 different possible scenarios (A to G) of interaction-associated mutants conservation. Each scenario is then illustrated by a heatmap of the corresponding genes IFE values in all conditions (only the significant IFE are shown) along with the IFE profile of one example gene found in the scenario (Significant IFE: plain color, non-significant IFE: transparent color). Heatmaps have been generated in R (version 3.6.1; https://www.r-project.org/) and RStudio (version 1.0.143; https://www.rstudio.com/products/rstudio/older-versions/) using the package ComplexHeatmap (version 2.0.0; https://bioconductor.org/packages/release/bioc/html/ComplexHeatmap.html / Gu Z, Eils R, Schlesner M (2016). “Complex heatmaps reveal patterns and correlations in multidimensional genomic data.” *Bioinformatics*. doi: 10.1093/bioinformatics/btw313.)

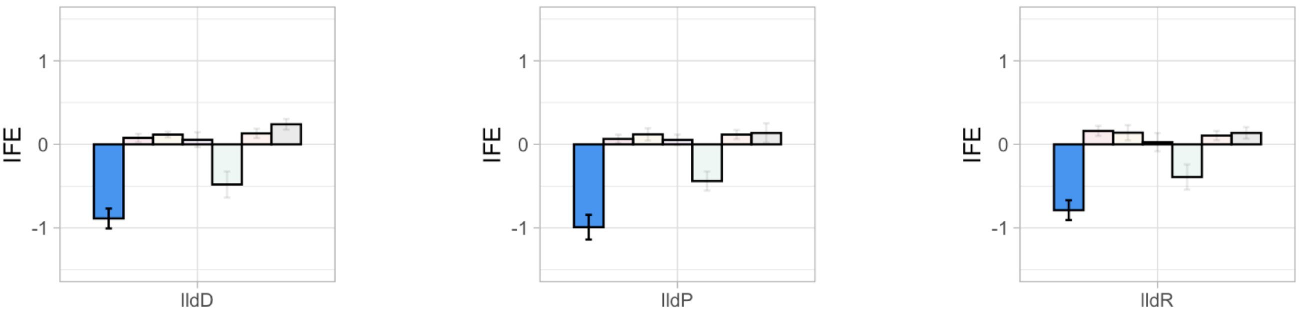

<u>Supplementary figure 7: IFE profiles of lactate metabolism genes.</u>

Maintained and **Dropped 2-species interaction-associated mutants** in 3-species cultures

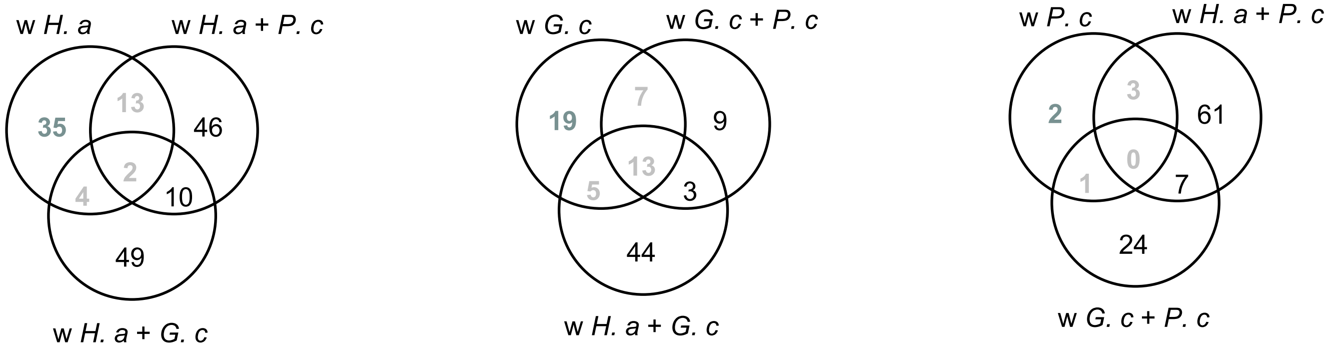

Emerging interaction-associated mutants and **2-species maintained interaction-associated mutants** in 3-species cultures

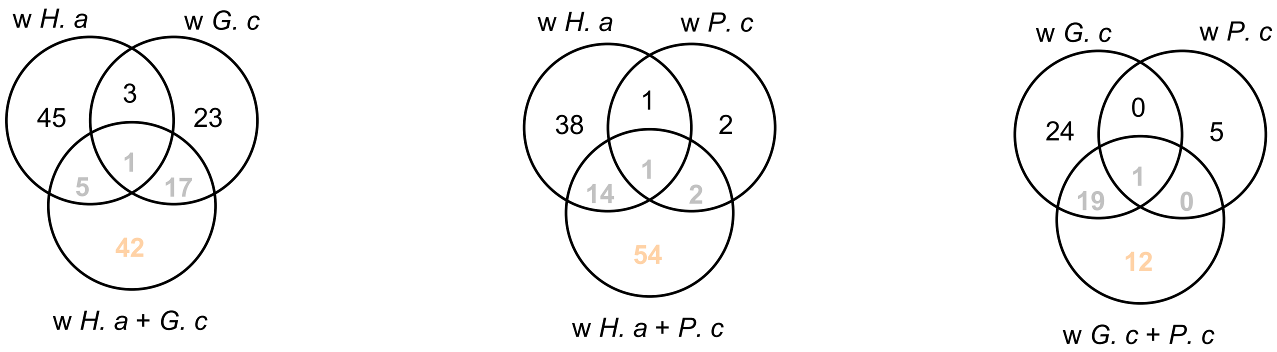

H. a: *Hafnia alvei*
G. c: *Geotrichum candidum*
P. c: *Penicillium camemberti*

Supplementary figure 8: Condition specific comparison of interaction-associated mutants for 2 and 3-species conditions

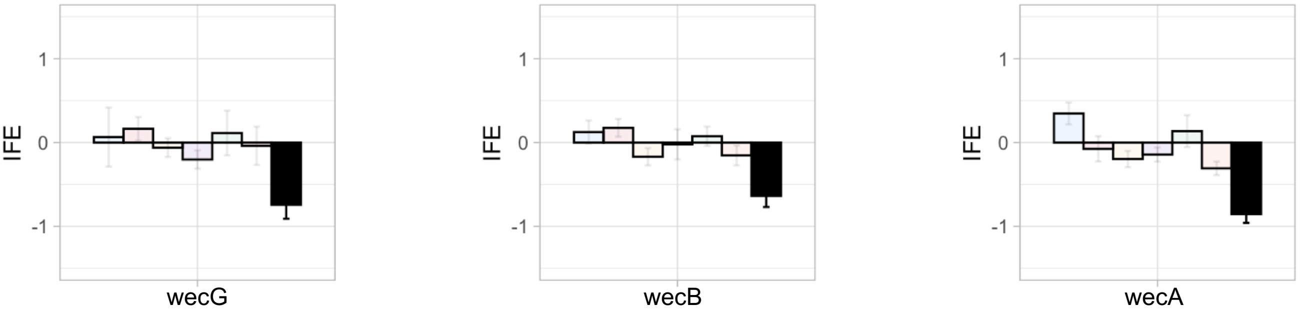

Supplementary figure 9: IFE profiles of Enterobacterial Common Antigen (EAC) genes

3-species dropped interaction-associated genes and **3-species maintained interaction-associated genes** in 4-species cultures

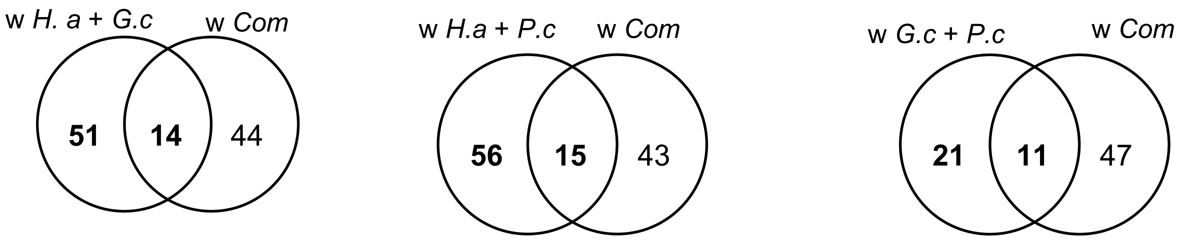

H. a: *Hafnia alvei*
G. c: *Geotrichum candidum*
P. c: *Penicillium camemberti*
w Com= Hafnia alvei + Geotrichum candidum + Penicillium camemberti

Supplementary figure 10: Condition specific comparison of interaction-associated mutants for 3 and 4-species condition

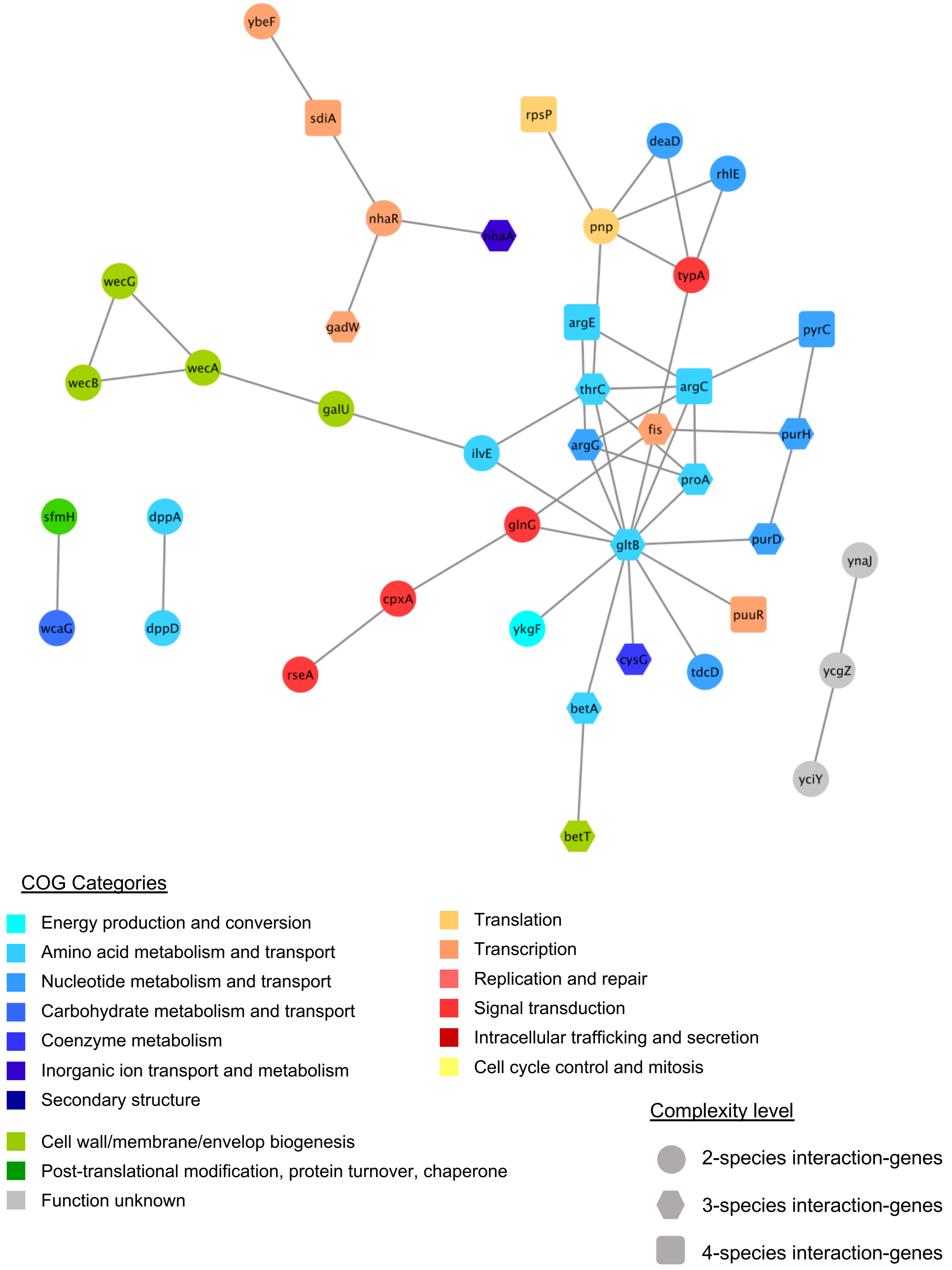

Supplementary figure 11: Functional network of the 4-species interaction-genes and their origin
STRING **n**etwork of the genes (Nodes) associated with interactions in the 4-species condition. Edges connecting the genes represent both functional and physical protein association and the thickness of the edges indicates the strength of data support (minimum required interaction score: 0.4 – medium confidence). Nodes are colored based on their COG annotation and the shape of each node represents the level of community complexity the 4-species interaction-mutants originate from.

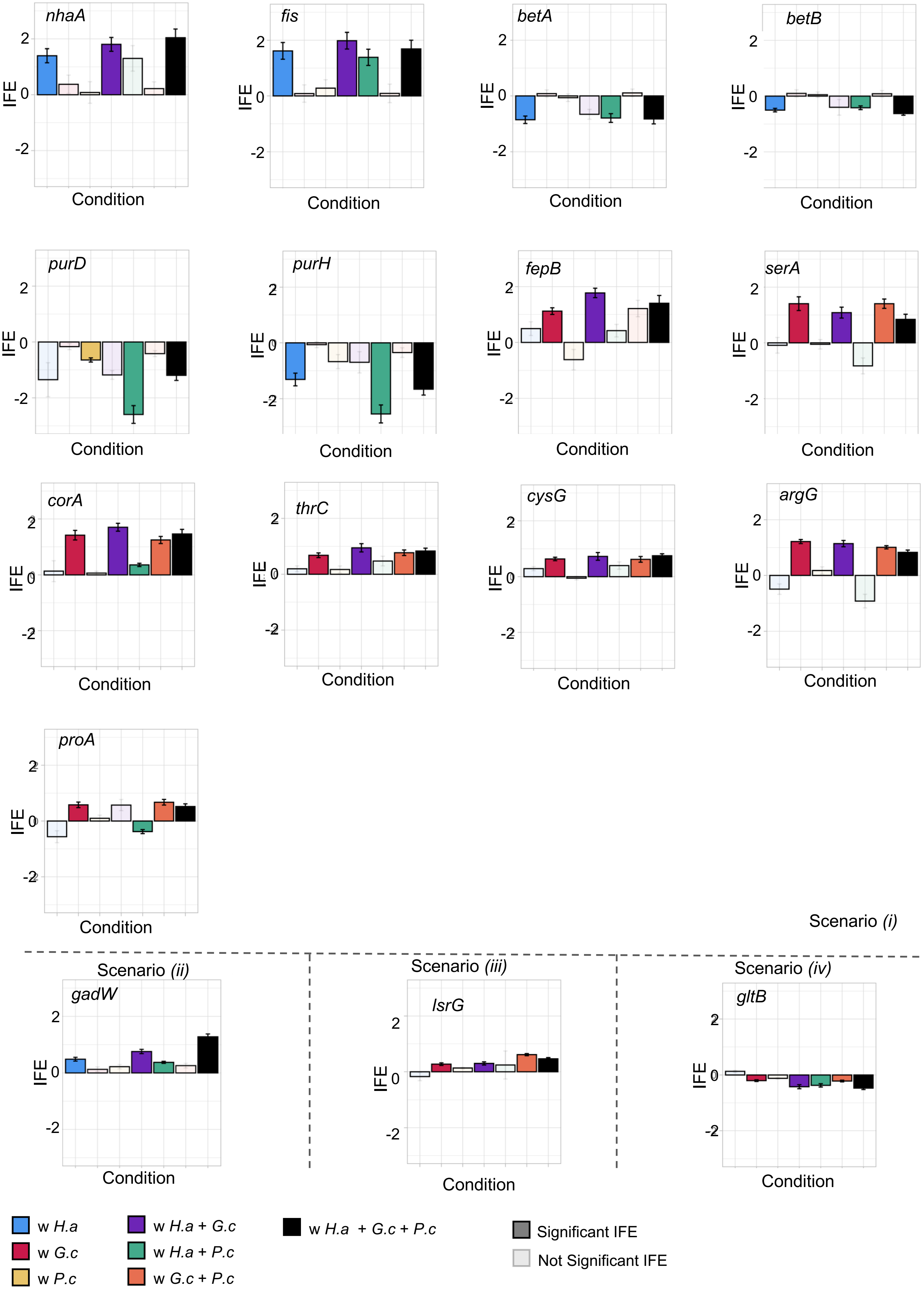

Supplementary figure 12: IFE profiles of the 16 genes associated with the 2-species interaction mutants maintained up to 4-species

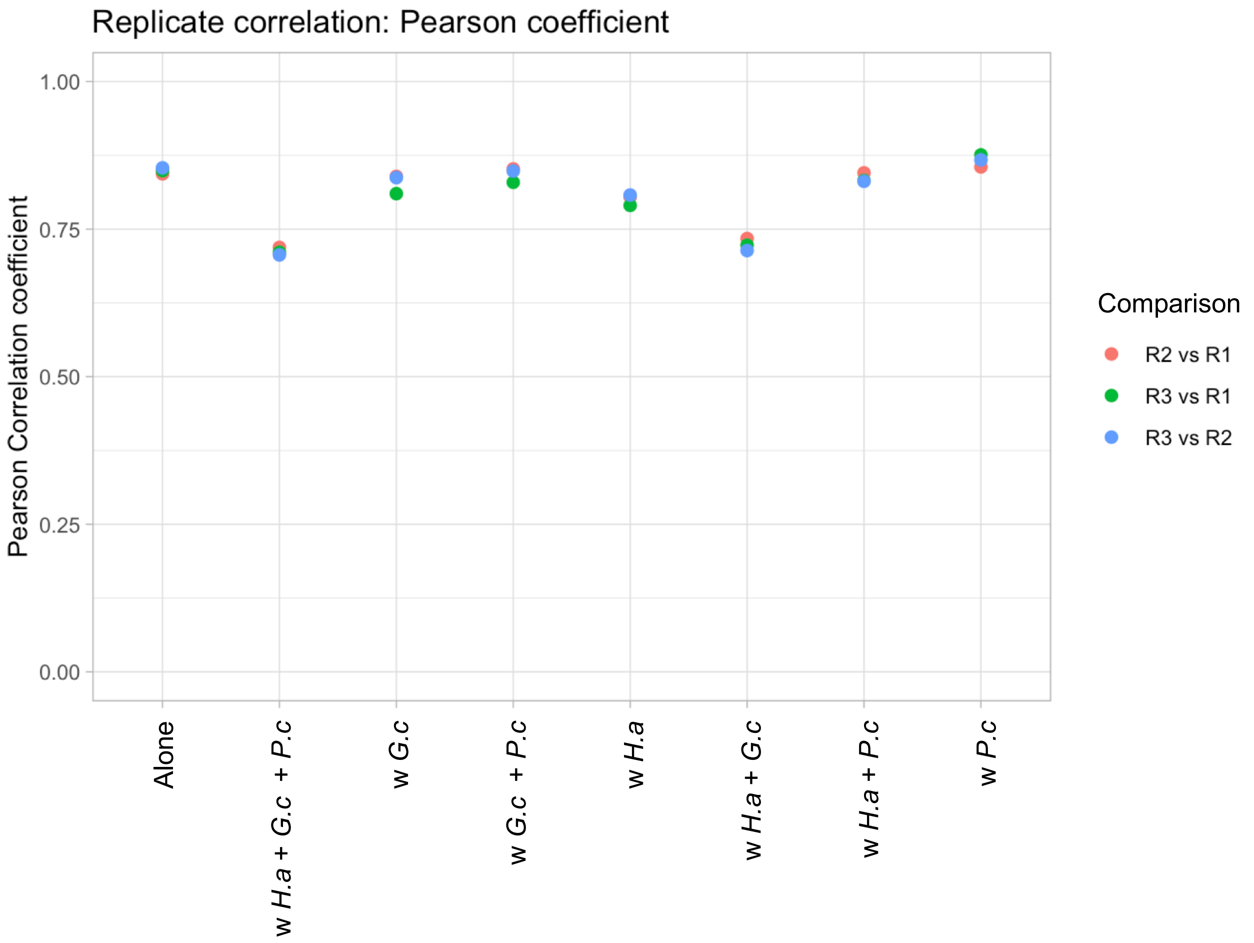

Supplementary figure 13: Pearson correlation of gene fitness across replicates

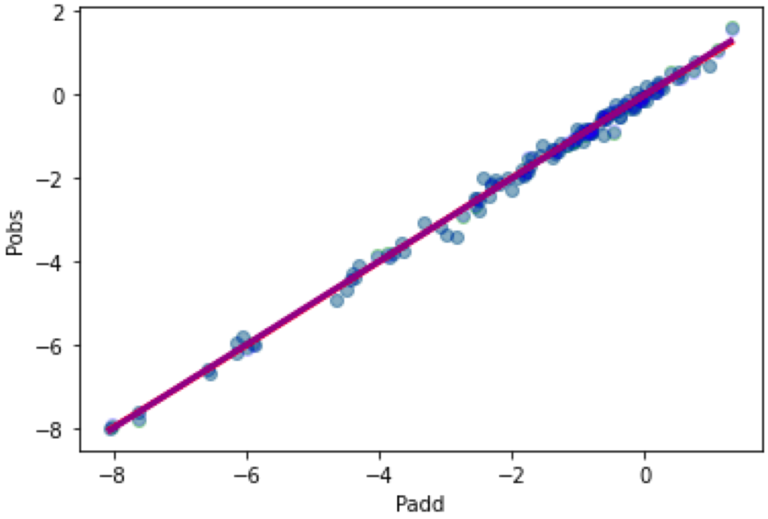

Supplementary figure 14: Non-linearity analysis of IFE in the Epistatis model

Predicted IFEs from an additive model (Padd) plotted against the Observed IFEs (Pobs) for the 16 genes associated with interactions from 2-species up to 4-species condition. No deviation from the identity line indicate that the IFE combine linearly and that there is no need for non-linearity correction[17].
